# Co-Delivery of siRNA and Chemotherapeutic Drug Using 2C5 Antibody-Targeted Dendrimer-Based Mixed Micelles for Multidrug Resistant Cancers

**DOI:** 10.3390/pharmaceutics14071470

**Published:** 2022-07-15

**Authors:** Satya Siva Kishan Yalamarty, Nina Filipczak, Xiang Li, Tanvi Vinod Pathrikar, Colin Cotter, Vladimir P. Torchilin

**Affiliations:** 1 Center for Pharmaceutical Biotechnology and Nanomedicine, Northeastern University, Boston, MA 02115, USA; yalamarty.s@northeastern.edu (S.S.K.Y.); nin.filipczak@northeastern.edu (N.F.); pathrikar.t@northeastern.edu (T.V.P.); cotter.c@northeastern.edu (C.C.); 2 State Key Laboratory of Innovative Drug and Efficient Energy-Saving Pharmaceutical Equipment, Jiangxi University of Chinese Medicine, Nanchang 330004, China; xiang.li@jxutcm.edu.cn

**Keywords:** multidrug resistance, P-gp, immunomicelles, siRNA, 2C5 antibody, dendrimers, cancer

## Abstract

Multidrug resistance (MDR) observed in tumors significantly hinders the efficacy of chemotherapy. Downregulation of efflux proteins, such as P-glycoprotein (P-gp), using small interfering RNA (siRNA) can be an effective way to minimize the resistance in tumors. In this study, monoclonal antibody 2C5 (mAb 2C5)-PEG_7k_-DOPE conjugates were post-inserted into the mixed dendrimer micelles containing generation 4 (G4) polyamidoamine (PAMAM)-PEG_2k_-DOPE and PEG_5k_-DOPE. The inherent amphiphilic nature of DOPE conjugates causes the copolymers to self-assemble to form a micelle, which can encapsulate hydrophobic chemotherapeutic drugs in its core. The siRNA electrostatically binds to the cationic charges on the G4 PAMAM dendrimer. The tumor-specific mAb 2C5 on the surface of these nano-preparations resulted in improved tumor targeting. This active targeting to tumors can cause increase in the drug and siRNA accumulation at the tumor site, and thereby minimizing the off-target effects. The micelles were shown to have higher cellular association and effectiveness in vitro. The immunomicelle preparation was also tested for cytotoxicity in breast (MDA-MB-231) and ovarian (SKOV-3TR) MDR cancer cell lines.

## 1. Introduction

Cancer has remained a leading cause of death worldwide for a few decades now [1]. Chemotherapies to treat cancers exist in the market but their therapeutic effectiveness remains questionable. The major barrier to the development of effective cancer treatment therapeutics can be attributed to the development of simultaneous resistance to multiple drugs [2]. Multidrug resistance (MDR) is a common phenomenon for chemotherapeutic drug failure in treating the cancer. Several factors are responsible for MDR in tumor cells, mainly cellular factors, such as altered molecular targets, increased drug metabolism and genetic defects (polymorphism, reduced apoptosis, over expression of efflux pumps, improved DNA repair pathways), and physiological factors, such as cell–cell interaction, higher interstitial fluid pressure, a low pH environment, regional hypoxia in the tumor, irregular tumor vasculature, and presence of cancer cells that are present in areas difficult to penetrate [3]. Resistance to conventional chemotherapies would result in the application of increased dosages, resulting in increased toxicities and decreased patient quality of life. Counteraction of MDR is an effective method for cancer treatment.

One of the mechanisms that most commonly causes MDR is associated with an increased efflux of hydrophobic chemotherapeutic drugs, which is mediated by the energy-dependent ATP-binding cassette (ABC) transporters. Some of the transporters in this class include P-gp (P-glycoprotein), MRP1 (multidrug resistance-associated protein-1), BCRP (breast cancer resistance protein) and MXR (mitoxantrone resistance protein) [2,4,5,6]. These transporters have a broad range of specificities and can promote the efflux of several classes of xenobiotics [7]. Among these, the ABC class of transporters, P-gp tends to stand out, since they cause resistance to a broad variety of chemotherapeutic agents. P-gp, the protein encoded by the MDR1 gene, is also expressed in normal transport epithelium of the liver, kidney, and gastrointestinal tract that detoxifies and protects the normal tissues from xenobiotics [8,9]. However, in tumor cells, an overexpression of P-gp results in intracellular reduction in chemotherapeutic drugs and their accumulation [10,11].

Selective suppression of the MDR1 gene in tumor cells can effectively reverse multidrug resistance. The downregulation of P-gp via cancer specific pathways has been developed to retain the constitutive expression of P-gp in normal cells [12]. Several small molecule P-gp inhibitors, such as ketoconazole, verapamil, and clarithromycin, are available on the market. However, these small molecule drugs that block P-gp come with side effects and do not downregulate the protein expression at the gene level [13]. Current research has shown that the endogenous tool, small-interfering RNA (siRNA), can effectively downregulate P-gp and lead to MDR reversal [14]. Furthermore, this strategy of transcriptional repression is not only highly specific but also aids in the prevention of P-gp expression during the progression of disease. Several challenges are involved in siRNA delivery to the cancer cells, including poor cellular uptake, rapid clearance, non-ideal biodistribution and instability [15,16].

In this study, we prepared mixed dendrimer micelles (MDM) using an amphiphilic triblock copolymer, which was G4 poly(amidoamine) (PAMAM) dendrimer based. The 1,2-dioleoyl-sn-glycero-3-phosphoethanolamine-conjugated poly(ethylene glycol)5k (PEG_5k_-DOPE) and PAMAM-PEG_2k_-DOPE can self-assemble into micelles due to their amphiphilic nature. The cationic charges on the PAMAM polymer can electrostatically bind to the negatively charged phosphate groups on siRNA, while the hydrophobic chemotherapeutic drugs can be loaded into the lipid core of the micelles. This platform enables a simultaneous delivery of both the chemotherapeutic drug and siRNA to the cancer cells and exploits their synergistic effects. Our previous research showed that when these micelles were loaded with doxorubicin (DOX) and siMDR1 (siRNA specific to MDR1 gene), a significant downregulation of the membrane bound P-gp was observed in MDR cancer cells [17]. Traditionally, the nanoparticles are not specific to the tumor site and are cleared very quickly from the body. To improve specificity of the MDM, we take a targeted delivery approach [18,19]. To make it more specific for cancer cells, we conjugated a monoclonal antibody (2C5) to a PEG_7k_-DOPE polymer that was incorporated into the MDM in a previous study [20]. The antibody is specific to nucleosomes on the cancer cell surface, which resulted from apoptotically dying neighboring cells [21,22]. The 2C5-modified micelles can be made to be very specific to the tumor cells and lower the systemic toxicities [23,24,25]. Moreover, the MDM can be modified to suit several functions based on its composition to further improve the efficacy. The major advantage is the ease of preparation of the MDM due to their self-assembly of the copolymers.

Our previous studies showed that mAb 2C5-modified micelles loaded with DOX and siMDR1 improved cellular uptake, cytotoxicity, and enhanced anticancer effects towards A2780 ADR ovarian cancer in vivo [20]. Here, in this study, we want to further investigate the applicability of the platform in terms of its stability and efficacy with cancer cell lines such as MDA-MB-231 (triple negative breast cancer) and SKOV-3TR (ovarian cancer). We also wanted to check the shelf life of the formulation, which is a major determining factor affecting the robustness of the formulation.

## 2. Materials and Methods

### 2.1. Materials

1,2-dioleoyl-sn-glycero-3- phosphoethanolamine-N-(methoxy(polyethylene glycol)-5000) (PEG_5k_-DOPE), 1,2-dioleoyl-sn-glycero-3-phosphoethanolamine (DOPE) and 1,2-dioleyl-sn-glycero-3-phosphoethanolamine-N-(carboxyfluorescein) (carboxyfluorescein-DOPE) were purchased from Avanti Polar Lipids (Alabaster, AL, USA). PAMAM dendrimer with an ethylenediamine core, generation 4, 10% *w*/*w* solution in methanol (G(4)-D) was purchased from Sigma-Aldrich. NPC-PEG-7K-NPC (pNP-PEG_7k_-pNP) and NPC-PEG-2K-NPC (pNP-PEG_2k_-pNP) were purchased from Laysan Bio (Arab, AL, USA). Bovine serum albumin (BSA), dimethylformamide (DMF), methanol, protease inhibitor cocktail, FAM-labeled negative siRNA, Ambion^TM^, SuperSignal^TM^ West Pico PLUS Chemiluminescent Substrate, NovexTM 4–20% Tris-Glycine Mini Gels, WedgeWell^TM^ and Micro BCA assay kit were purchased from Thermo-Fisher Scientific (Waltham, MA, USA). siRNA targeting MDR-1 (siMDR-1) (5′- GGAAAAGAAACCAACUGUCdTdT-3′ (sense) [26] was purchased from GE Healthcare Dharmacon, (Lafayette, CO, USA). Nuclease-free water was purchased from Qiagen (Germantown, MD, USA). Doxorubicin HCl was purchased from LC Laboratories (Woburn, MA, USA). The CellTiter-Blue^®^ and CellTiter-Glo^®^ Cell Viability Assays were purchased from Promega (Madison, WI, USA).

Monoclonal antibody mAb 2C5 (Γ2a;κ) was obtained from Harlan Labs using the 2C5E3 hybridoma cell line from our lab. Poly-L-lysine hydrobromide (MW 30,000–70,000) (P2636-25 MG) was purchased from Sigma. Calf thymus nucleohistone (LS003011) was acquired from Worthington Biochemical Corporation.

Doxorubicin-resistant human breast adenocarcinoma cell line (MDA-MB-231 ADR) and paclitaxel-resistant human ovarian adenocarcinoma cell line (SKOV-3 TR) were purchased from the American Type Culture Collection (ATCC). Dulbecco’s Modified Eagle’s Media (DMEM), fetal bovine serum (FBS) and penicillin-streptomycin solution were obtained from CellGro (Manassas, VA, USA). The trypan blue solution was obtained from Hyclone (Logan, UT, USA). Molybdenum blue spray reagent, anhydrous chloroform, RIPA buffer, Dragendorff reagent and phosphate buffered saline (PBS) were purchased from Sigma-Aldrich (St. Louis, MO, USA). HEPES was purchased from MP Biomedicals (Solon, OH, USA). Primary anti-Pgp antibody and horseradish peroxidase-IgG antibodies (goat anti-rabbit) were purchased from Abcam (Cambridge, MA, USA). Anti-β-actin antibody and horseradish peroxidase-IgG antibodies (goat anti-mouse) were purchased from Santa Cruz Biotechnology (Dallas, TX, USA). PVDF membrane (iBlotTM 2 Transfer Stacks, PVDF) was obtained from Invitrogen (Carlsbad, CA, USA).

### 2.2. Methods

#### 2.2.1. Preparation of Dox-Loaded MDM and 2C5-Modified Micelle

The micelles were prepared according to our previously mentioned paper [17,18]. The conjugates along with doxorubicin (4% *w*/*w* of the lipid) were pipetted into a glass test tube to form a thin film and later hydrated with HEPES glucose (BHG) buffer with siRNA. The 2C5-PEG7K-DOPE conjugate was post inserted into the micelles via incubation at 37 °C for 1 h. The antibody to polymer ratio was 1:100 in the antibody-modified micelles.

For imaging purposes, PEG5K-DOPE in the micelles were replaced with carboxyfluorescein-tagged DOPE.

#### 2.2.2. Particle Size, Polydispersity, Zeta Potential Measurement and Drug Concentration Analysis

Size, polydispersity, and zeta potential of the nanoparticles were evaluated by dynamic light scattering (DLS) in a Zetasizer Nano ZS 90 (Malvern Instruments, Malvern, UK). Using a reverse-phase HPLC method, we quantified the concentration of doxorubicin in the polymeric nanoparticles using a Hitachi Elute LaChrome HPLC system by first dissolving MDM nanoparticles in acetonitrile [27].

#### 2.2.3. Surface Morphology

Transmission electron microscopy (TEM) (JEOL USA, Inc., Peabody, MA, USA) was used to study the surface morphology of the MDM nanoparticles. Diluted (in saline) and undiluted nanoparticles were placed on a copper grid and dried at RT. This step was followed by staining with 2% uranyl acetate and dried at RT as well. Excess sample was removed from the grid, and suitable images were taken at different magnifications.

#### 2.2.4. Homolysis of Dendrimer-Based Nanoparticles

The blood was collected and homogenized, and later centrifuged at 500× *g* for 5 min. The supernatant was discarded; later the erythrocytes were mixed with 1mL saline and centrifuged at 500× *g* for 5 min. This washing step was repeated 2 more times. The erythrocytes were then resuspended in saline at a concentration of 2% (*v*/*v*). An amount of 100 μL of formulation was added into a 96 well plate. In addition, 1% Triton X was used as a positive control. Then, 100 μL of 2% erythrocyte solution was added to each well. Samples were incubated for 1 h under agitation at 37 °C, and centrifuged at 500 g for 10 min. The supernatant from each well was loaded into a fresh 96 well plate and absorbance measured (for released hemoglobin) using a spectrophotometer at 540 nm. Blank samples (without mixing with erythrocytes) of each concentration of MDM formulation were also evaluated on the spectrophotometer for background signal. The % hemolysis was calculated by considering the absorbance observed for 1% Triton X treatment as 100% hemolysis.

#### 2.2.5. Two-Dimensional Cell Culture Preparation

Both cancer cell lines MDA-MB-231 and SKOV-3TR were cultured in in DMEM 4.5 g/L glucose. All media was supplemented with 10% FBS and antibiotics (100 IU/mL streptomycin). Both cell lines were cultured at 37 °C with 5% CO_2_. To maintain the MDR effect, the cells were resuspended in media containing 100 nM dox HCl after each passage [28].

#### 2.2.6. Cellular Uptake of MDM Nanoparticles in Monolayer

Cells (2 × 10^5^) for both cell lines were seeded in a 6-well plate and allowed to grow overnight. Cells were incubated with the nanoparticles containing doxorubicin and FAM-labeled siRNA for 15, 30, 45 and 60 min in serum complete media. Later, the cells were detached using trypsin and washed with PBS (pH 7.4) for three times and resuspended in 200 μL of PBS. The cellular association of nanoparticles was evaluated using flow cytometry (Beckton Dickinson FACS CaliburTM, Piscataway, NJ, USA) by measuring the fluorescence of FAM-siRNA. The fluorescence signal was excited using a 488 nm laser and the emission was recorded using a standard FL-1 filter (λ = 530 nm) and FL-3 filter (λ = 585 nm). A total of 10,000 gated living cell events were collected.

#### 2.2.7. P-gp Downregulation in Monolayers Using Western Blot

Both cell lines were cultured in 6-well plates (approximately 500,000 cells/well), treated with nanoparticles containing siRNA for 1 and 4 h. The cells were then harvested and lysed in RIPA buffer (150 mM NaCl, 50 mM Tris, 1% NP-40, 0.5% sodium deoxycholate, 0.1% SDS, pH 7.4), supplemented with protease inhibitor cocktail, sodium orthovanadate (1 mM), and PMSF (1 mM). The whole-cell lysates were normalized for protein content using Micro BCA assay. Proteins (20 μg) were resolved by SDS-PAGE, transferred to a PVDF membrane, and probed with anti-Pgp antibody and anti-β-actin antibody. Following treatment with secondary horseradish peroxidase-IgG antibodies, goat anti-rabbit and goat anti-mouse, the blots were incubated in chemiluminescent solution for 5 min and then imaged on ChemiDocTM XRS + System (Bio-Rad, Hercules, CA, USA). Densitometry analysis was performed with Image J and Microsoft Excel.

#### 2.2.8. Cellular Association by Fluorescence Imaging

A total of 50,000 cells (MDA-MB-231and SKOV-3TR) were seeded in 24-well plate ibidi^®^ plates and left overnight to adhere. The following day, the cells were treated with carboxyfluorescein-tagged micelles. Both 2C5-modified and non-modified micelles were tagged with the fluorophore and cellular association was evaluated in a time dependent manner. After treatment, the cells were washed twice with PBS buffer and later stained with Hoechst for 10 min and washed twice with PBS before imaging (Keeyence BZ-X 700 all in one microscope).

#### 2.2.9. Cytotoxicity of Nanoparticles in a Monolayer Model

A total of 5000 cells of both the cell lines were seeded in 96-well plates and incubated overnight in an incubator. Afterwards, the cells were treated with varying concentrations of doxorubicina and nanoparticles containing Dox (concentration range: 1.1 μM to 9 μM) and siRNA (concentration range: 62.5 nM to 500 nM per well). The highest concentration for this experiment was 500 nM of siRNA + 9 μM of Dox and then serially diluted. For all groups, the cells were treated with a respective formulation for 1 or 2 h, followed by washing and incubation. Cell viability was measured after 24 or 48 h incubation using a Cell Titer-Blue^®^ cell viability assay by following the manufacturer’s protocol.

#### 2.2.10. Statistical Analysis

All data are presented as mean ± SD, *n* = 3. The data were analyzed using one and two-way ANOVA multiple comparisons with GraphPad prism 9.3.1 (350) for Macintosh (GraphPad Software, Inc., San Diego, CA, USA). Any *p* value < 0.05 was considered statistically significant and denoted as *.

## 3. Results

### 3.1. Stability of MDM

The stability of MDM was tested in 4 °C storage conditions. The prepared formulation was stored at 40 °C right after the zero timepoint was obtained. Table 1 shows the size and zeta potential of the prepared MDMs at multiple timepoints. There were no significant changes in the size and zeta potential during the study, indicating a stable formulation. The data in Table 1 show that the antibody-modified MDM were stable until 20 days at 40 °C.

### 3.2. Imaging of Micelles Using TEM

**Figure 1:**
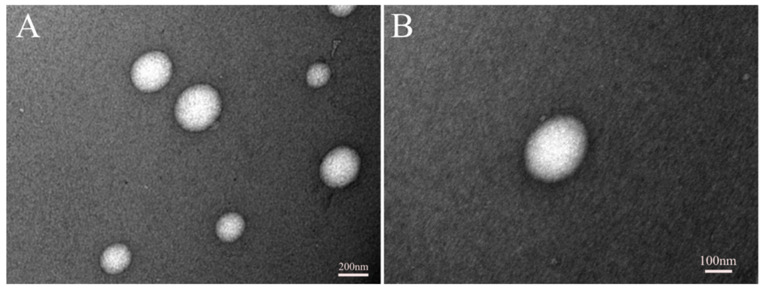
TEM Images of 2C5-modified mixed dendrimer micelles (2C5-MDM-DR), (**A**) 10,000× magnification, (**B**) 20,000× magnification.

The 2C5 antibody-modified micelles were prepared according to our previously published paper [17]. The prepared micelles were imaged with a transmission electron microscope (TEM). Figure 1 shows the images of the micelles at 10,000× and 20,000× magnification in panels A and B, respectively. The images obtained via TEM corresponded to the size data published in a previous study [17].

### 3.3. Hemolysi

**Figure 2:**
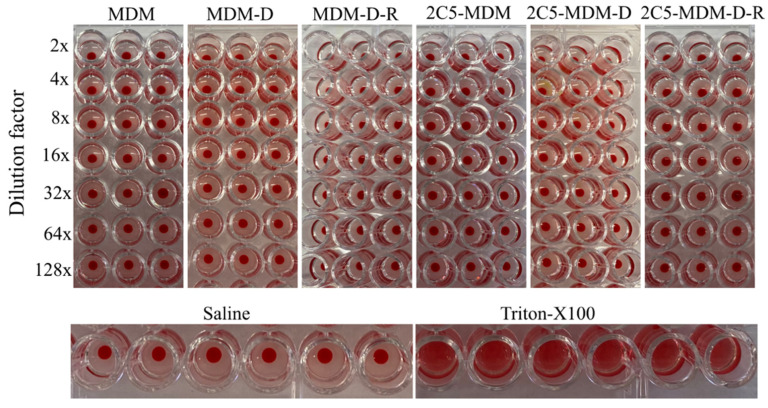
Hemolysis data depicting the safety of the micelles prepared. The stock nanoparticles in this experiment had 1.8 mg/mL of PAMAM.

Hemolysis is the destruction of red blood cells (RBCs). The 2C5-modified MDM along with non-modified micelles were tested at different dilution factors, (Figure 2). Treatment with 2C5-modified micelles and non-modified micelles produced no hemolytic activity. Hemolysis between 10 and 25% is the standard for hemolytic activity. Any value below 10% of hemolysis can be considered non-hemolytic [29]. Both negative (saline) and positive controls (Triton X) were used to show the difference between hemolytic activity and non-hemolytic activity. The blood used in the experiment was from healthy female mice (C57BL/6) blood.

### 3.4. Cellular Association of 2C5-Modified MDM with Cancer Cells

**Figure 3:**
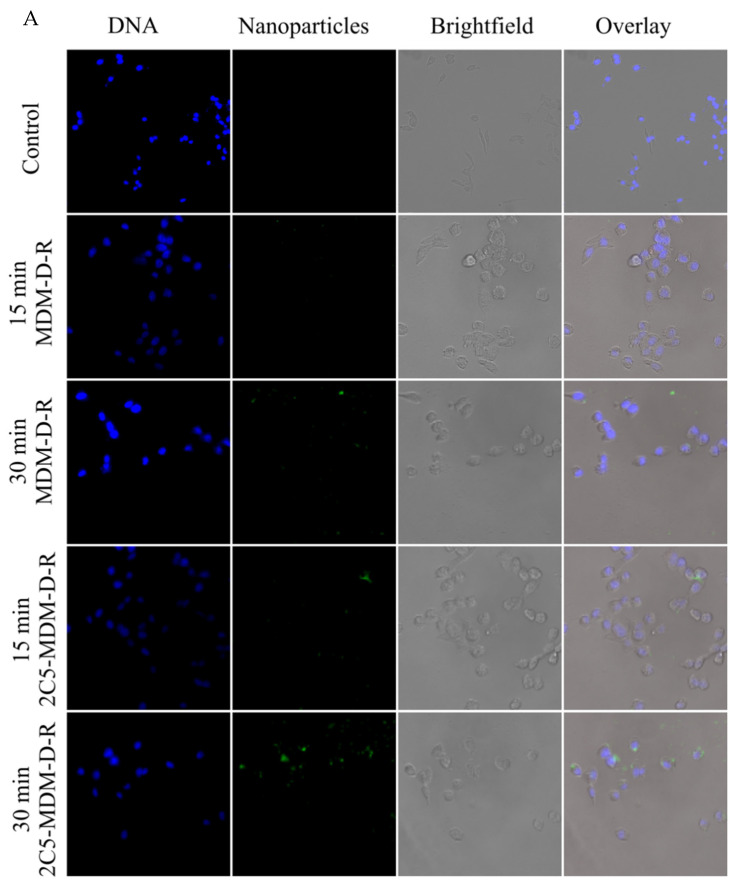

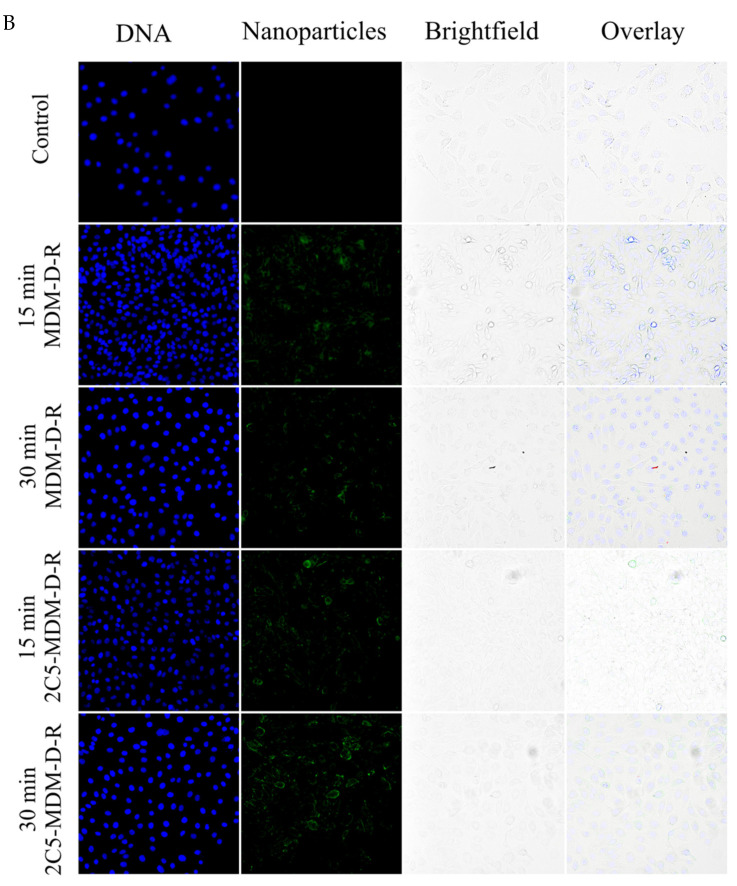
Cellular association of 2C5-modified and non-modified nanoparticles. The figure shows the Hoechst-stained nuclei with carboxyfluorescein-labelled nanoparticles, along with brightfield and overlay images. The carboxyfluorescein-labelled nanoparticles were incubated with (**A**) MDA-MB-231 cells and (**B**) SKOV-3TR cells for 15- and 30-min to observe a greater cellular association (greater green fluorescence) in the 2C5-modified dendrimer micelles in comparison to non-modified micelles. (Magnification 40×).

The superior cellular association of 2C5 antibody-modified micelles was observed via fluorescence imaging of cancer cells treated with nanoparticles. For this study, we treated MDA-MB-231 and SKOV-3TR cells with carboxyfluorescein-tagged micelles for 15 and 30 min and imaging was performed after staining the nuclei with Hoechst dye (blue color). To prove the 2C5-modified micelles have a higher association with the cancer cells, we prepared micelles that contained carboxyfluorescein-tagged DOPE lipid (5% of total moles lipid). This green dye can be observed in both 15- and 30-min time points of 2C5-modified micelles whereas, the non-modified ones show faint association in their 30-min time point (as shown in Figure 3A,B). Furthermore, SKOV-3TR cells show a slightly higher association with the nanoparticles than the MDA-MB-231 cells. It should also be noted that in a closed in vitro system, the time points of treatment are essential to decipher the difference between a targeted and non-targeted system. The non-targeted micelles when incubated long enough would show a similar effect in comparison to the targeted (2C5-modified) micelles. Hence, shorter time points of 15- and 30-min were chosen. Bright field imaging was used to show the morphology of the cancer cells.

### 3.5. Cellular Uptake by FACS

**Figure 4:**
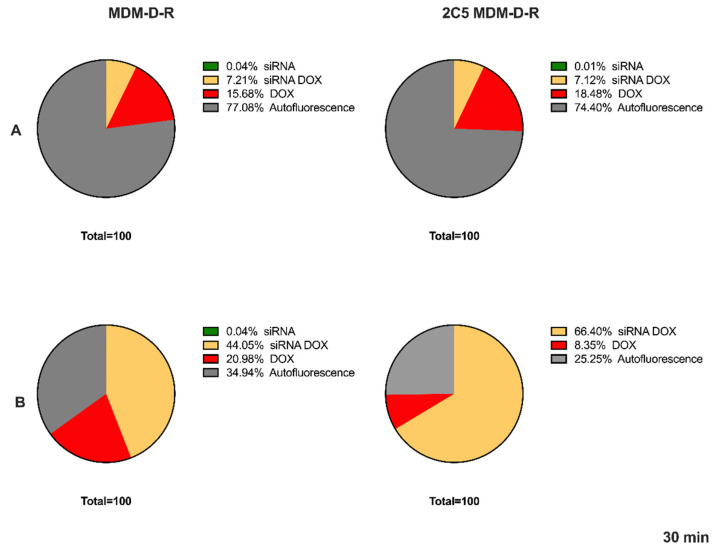
Cellular update data using FACS after 30 min of treatment, (**A**) Skov-3TR and (**B**) MDA-MB-231.

Flow cytometry was used to study the cellular internalization of the 2C5-modified and non-modified micelles (Figure 4). The cells treated with the respective formulations for 30 min showed the difference in targeted and non-targeted formulations. Figure 4 shows the results for both the MDA-MB-231 cell line and SKOV-3TR cell line. A higher fluorescence was observed in both cell lines of the targeted group in comparison to the non-targeted group. Initially multiple time points were performed, and we did not notice any difference in the uptake beyond the 30 min timepoint. The reason the longer timepoints have not shown any difference between targeted and non-targeted is because in a closed in vitro system given enough time, both targeted and non-targeted can act similarly. Beyond that, in an in vitro system, there are no physiological barriers involved besides the cell membrane; hence, time plays a key role in differentiating targeted and non-targeted formulations using this study. In shorter timepoints, there was not enough uptake to show the difference between the two groups.

### 3.6. Downregulation of Membrane Bound P-gp

**Figure 5:**
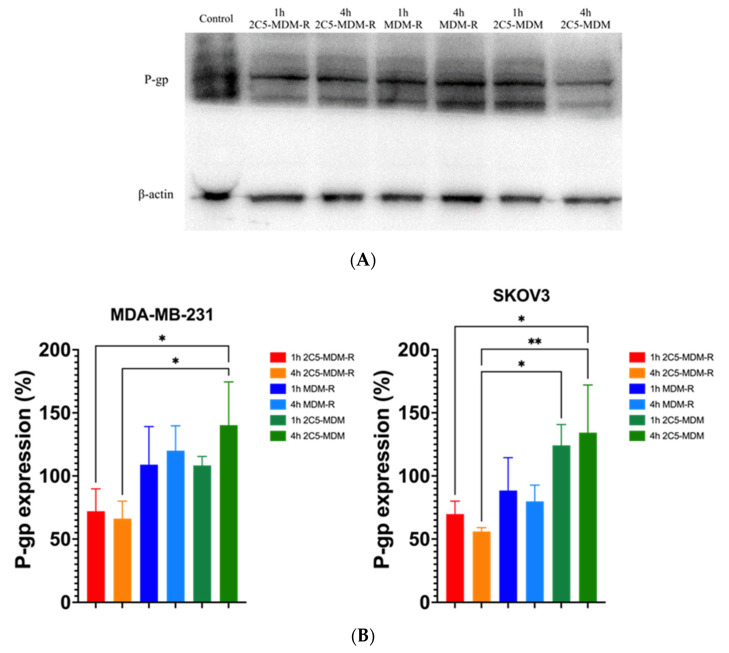
P-gp downregulation measured using Western blot; (**A**) representative Western blot gel image, (**B**) densitometry analysis with the 2C5-modified micelles in both MDA-MB-231 and Skov-3TR cells. Results indicate ± SD (*n* = 3), and significance was calculated with one-way ANOVA comparisons. ** *p* ≤ 0.01, * *p* ≤ 0.05.

The effect of siRNA in downregulating the P-gp expression was studied using a Western blot analysis. Figure 5 shows the level of P-gp downregulation in 2C5-modified MDM after 1- and 4-h treatments in both MDA-MB-231 and SKOV-3TR cell lines. There was a significant downregulation of P-gp expression in the formulation loaded with siRNA versus the siRNA control. Although visually there is a difference between 2C5-modified MDM and non-antibody modified MDM, there is no statistical difference between them. At 4-h, treatment with 2C5-modified MDM with dox and siRNA show a significantly lower P-gp expression than the 1 h treated group, indicating the time dependent uptake of the formulation into the cells and a downregulation of P-gp. A concentration of 500 nM of siRNA was used in both 2C5-modified and non-antibody modified MDM to treat both the cell lines. A lower expression was observed with the 2C5-modified formulation over the non-targeted formulation because of its targeting capability.

### 3.7. Cytotoxicity in Monolayer Model

**Figure 6:**
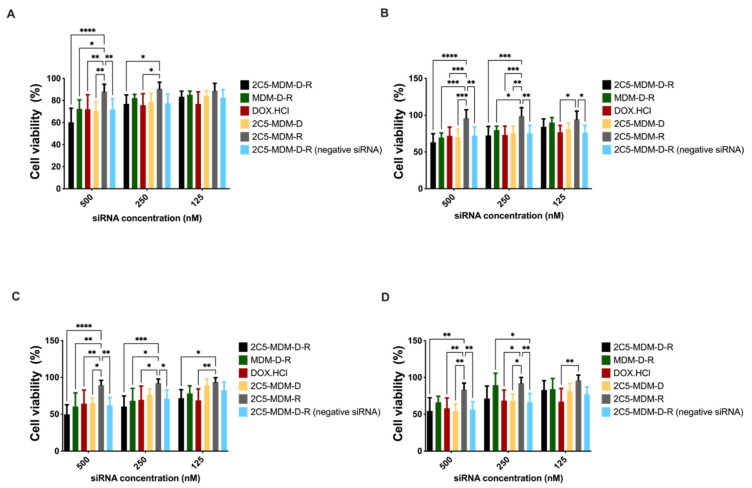
Cell titer blue (CTB) assay of 2D monolayer cytotoxicity study of MDA-MB-231, (**A**): 1 h of treatment and 23 h of recovery, (**B**): 2 h treatment and 22 h of recovery, (**C**): 1 h treatment and 47 h of recovery, (**D**): 2 h treatment and 46 h of recovery. Results indicate ± SD (*n* = 3), and significance was calculated with two-way ANOVA comparisons. **** *p* ≤ 0.0001, *** *p* ≤ 0.001, ** *p* ≤ 0.01, * *p* ≤ 0.05.

**Figure 7:**
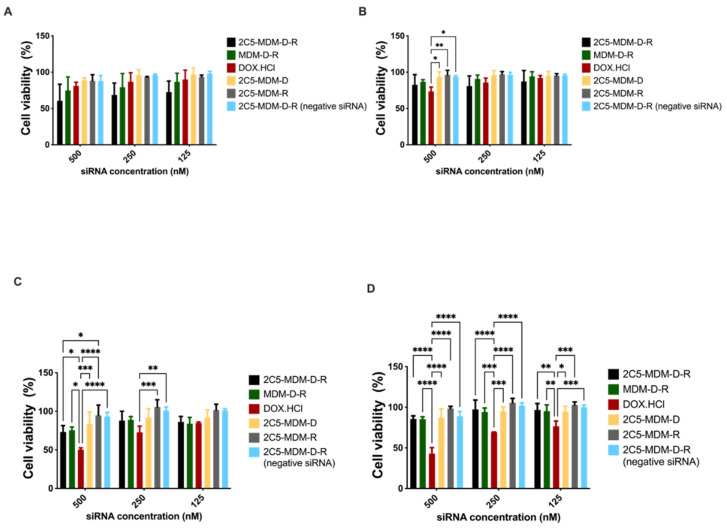
Cell titer blue (CTB) assay of 2D monolayer cytotoxicity study of Skov-3TR, (**A**) 1 h of treatment and 23 h of recovery, (**B**) 2 h treatment and 22 h of recovery, (**C**) 1 h treatment and 47 h of recovery, (**D**) 2 h treatment and 46 h of recovery. Results indicate ± SD (n = 3), and significance was calculated with two-way ANOVA comparisons. **** *p* ≤ 0.0001, *** *p* ≤ 0.001, ** *p* ≤ 0.01, * *p* ≤ 0.05.

The cell viability in the monolayer model was assayed with cell titer blue (CTB). The assay was performed on MDA-MB-231 and SKOV-3TR cells. Cells were treated with different dendrimer formulations and doxorubicin at several concentrations. The treatment was conducted for 1 or 2 h before replacing the media. The plates were then incubated for 23 h or 46 h at 37 °C for recovery. After the incubation, CTB reagent was added to evaluate the mitochondrial function in the viable cells. The concentration range from 500 nM to 125 nM of siRNA was used in both the cell lines. Figure 6A,B show the MDA-MB-231 cells’ viability with 1-h and 2-h incubation followed by 23- and 22-h recovery, respectively. In addition, 500 nM concentration of siRNA showed significant cytotoxicity. Similarly, Figure 6C,D show the cytotoxicity results of MDA-MB-231 cells with 1-h and 2-h treatment, followed by 47 and 46 h of recovery. The targeted formulation (2C5-MDM-D-R) showed a higher cytotoxicity in comparison to the other groups in all the incubation times for the MDA-MB-231 cell line. Figure 7A,B present the cytotoxicity results of SKOV-3TR cells with 1-h and 2-h incubation with 23 and 22 h of recovery, respectively. Similar to the MDA-MB-231 cells, 500 nM concentration showed higher cytotoxicity in SKOV-3TR cells as well. Figure 7C,D are the cytotoxicity results of 1-h and 2-h incubation timepoints with 47 and 46 h of recovery. Similar to MDA-MB-231, SKOV-3TR also showed a higher cytotoxicity in the targeted formulation (2C5-MDM-D-R). A higher cytotoxicity for DOX.HCl is observed in SKOV-3TR (Figure 7C,D) especially in the longer recovery times than the nano preparation. This could be an indication that the SKOV-3TR cell line might be more sensitive to doxorubicin in comparison to MDA-MB-231. Treatment groups 2C5-MDM-D, 2C5-MDM-R and 2C5-MDM-D-R (negative siRNA) were performed as controls for the 2C5-MDM-D-R formulation to evaluate the combination effect of siRNA and doxorubicin. All the control groups did not show any considerable cytotoxicity. Furthermore, it can be noticed that the cytotoxic effect of the formulation was better observed in longer recovery times. This is indicative of the time needed for the siRNA to downregulate P-gp.

## 4. Discussion

The current study describes the effectiveness of 2C5 antibody-modified mixed dendrimer micelles in vitro on triple negative breast cancer (MDA-MB-231) and ovarian cancer cell lines (SKOV-3TR). The mAb 2C5 was conjugated with pNP-PEG_7k_-DOPE as described previously [20]. Both the cell lines are known to be multidrug resistant cell lines [30,31,32]. The siRNA used in the formulation is specific to P-gp, which when downregulated increases the intracellular concentration of the drug, effectively increasing its efficacy. The micelles prepared are stable with respect to their size and zeta over the span of around 20 days at 4 °C. Table 1 shows the size and zeta of the formulation over the span of 20 days. The poly dispersity index (PdI) of the formulation in Table 1 indicates that the size of the micelles is uniform. Day 20 results indicate a slight shrinkage of the micelles over time. A slight drop in zeta potential was also observed at day 20. The TEM images in Figure 1 correspond to the size data noted in DLS studies. A size of around 150 nm was observed via TEM imaging. Both DLS and TEM studies were performed in 2C5-modified dendrimer micelles. The size and zeta results also correspond to those in our earlier studies [17,20]. The TEM images at 10,000× and 20,000× magnification was performed and are shown in Figure 1A,B, respectively.

Evaluating the hemolytic potential is very much necessary to address the biocompatibility of the nanoparticle formulation [33,34,35]. Hemolysis can cause phlebitis, vascular irritation, acute renal failure, anemia, jaundice and in some cases death [36]. We needed to make sure that the formulation we injected caused minimal irritation upon injection into vein. In this study, we evaluated the hemolytic potential of our dendrimer-based formulation at different concentrations and at a projected in vivo concentration (1.8 mg/mL of PAMAM dendrimer). From Figure 2, it is evident that the dendrimer-based formulation caused minimal to no hemolysis. Any hemolysis value below 20% has been considered a safe formulation [29]. Since the PAMAM-based formulation in this study showed hemolysis below 20% at all concentrations (Figure 2), adverse effects were not expected when injected to mice intravascularly.

Fluorescence imaging was performed in MDA-MB-231 and SKOV-3TR cells treated with 2C5-modified micelles containing FITC-DOPE (green color). Figure 3A,B show the fluorescence images of the cells treated with the nanoparticles containing FITC-DOPE. A total mole percentage of 5% FITC-DOPE was used in both 2C5-modified and non-modified micelles. We observed a higher cellular association of the targeted formulation at 30 min in comparison to the non-targeted in both MDA-MB-231 and SKOV-3TR cell lines. As a control Hoechst (blue color) was used to stain the nuclei of the cells, 0.2 mol% of the 2C5 antibody conjugate was used in the targeted micelle preparation. The nanoparticle view along with brightfield and overlay images clearly show a higher cellular association of 2C5-modifield micelles at 30 min.

The percentage or the density of the attached antibodies also could play a pivotal role in the targeting ability of the formulation [37]. The level of the 2C5 antibody was determined to be 0.1mol% based on our previous study [20]. In this study, we evaluated the ability of the formulation to target different cell lines. The cellular uptake studies performed in this study using FACS show the targeting ability of 2C5 antibody in both MDA-MB-231 and SKOV-3TR cell lines. Figure 4 shows the effect of cellular uptake of FAM-labelled siRNA and doxorubicin-loaded micelles. The data clearly show that the 2C5-modified micelles have improved cell uptake in comparison to the non-targeted micelle preparation. The cells were incubated with both targeted and non-targeted formulation for 15, 30 and 60 min and a significant cellular uptake was observed at the 30-min time point (Figure 4). The data from 15 and 60 min are not shown in the paper. Incubation time is very important in a cell uptake study performed to evaluate the targeting capability of the formulation. A shorter time-point was not enough to differentiate the targeting capability, whereas a longer time-point saturated both targeted and non-targeted formulations and interacted with the cells equally. Although 0.1 mol% was determined from our previous studies for cellular uptake, we have also evaluated 0.2 mol% in this current cell model. We have noticed a saturation of both targeted and non-targeted in these current cell lines at 0.2 mol% used, to observe the difference. We also noted a difference in the time-point from 0.1. to 0.2 mol%. In our previous cell model, cellular association was observed at the 1 h time-point and in the current study, we noticed the targeting ability at 30 min.

To evaluate the cellular internalization of the prepared mixed dendrimer micelles, we performed Western blots to evaluate the downregulation of membrane-bound P-gp. As shown in the Figure 5, both cell lines were evaluated to study the downregulation of P-gp, which is an indicator that the delivered siRNA is successfully escaping from the endosome and downregulating the MDR-1 gene. For this study, we prepared both 2C5 antibody targeted and non-targeted micelles without doxorubicin and treated both MDA-MB-231 and SKOV-3TR cell lines. The cell lines were treated for 1 h and 4-h, before allowing them to recover in fresh media overnight at 37 °C. The Western blotting analysis was performed the next day with siRNA loaded micelles, which showed a significant decrease in the P-gp expression in comparison to the non-siRNA loaded micelles. These data clearly show the therapeutic efficacy of siRNA-loaded nanoparticles. Moreover, the 2C5-modified nanoparticles decreased the P-gp expression in comparison to non-targeted micelles, proving the targeting capability of the antibody-modified micelles.

Finally, cytotoxicity studies in monolayer models were performed on MDA-MB-231 and SKOV-3TR cell lines. Figure 6 and Figure 7 show cytotoxicity data for MDA-MB-231 and SKOV-3TR cell lines, respectively. We treated the cells with our formulation for 1 and 2 h and changed the media and left the cells to recover for 23, 22, 47 and 46 h. Figure 6A,B show the MDA-MB-231 cells when treated for 1 and 2 h and recovered for 23 and 22 h, respectively. There was a dose–response relationship and a significant targeting effect of the 2C5-modified nanoparticles. Figure 6C,D are the cytotoxicity data for MDA-MB-231 cells after 1 and 2 h of treatment and 47 and 46 h of recovery. There was a dose–response relationship in both the treatment and recovery times. There was also a targeting effect observed in the longer recovery times as well. The control treatment groups 2C5-MDM-D, 2C5-MDM-R and 2C5-MDM-D-R (negative siRNA) have considerably lower cytotoxicity in comparison to 2C5-MDM-D-R. The cytotoxic response was also increased in the 2C5-MDM-D-R group in the longer recovery times. Cytotoxicity studies on monolayers were also performed on SKOV-3TR (Figure 7). As with MDA-MB-231 cells, we treated SKOV-3TR cells at the same dosage range. Figure 7A,B show the SKOV-3TR cells treated for 1 and 2 h and 23- and 22-h recovery times, respectively. A similar concentration range of siRNA has a relatively higher significant effect in MDA-MB-231 in comparison to SKOV-3TR cell line. Figure 7C,D show the SKOV-3TR cells treated for 1 h and 2 h with 47 and 46 h of recovery respectively. The targeted (2C5-MDM-D-R) and non-targeted (MDM-D-R) formulations have significantly higher cytotoxicity than the control groups. We can also observe that the doxorubicin groups in the longer recovery times for SKOV-3TR have a higher cytotoxicity than the other groups under study. This higher cytotoxicity can be due to the sensitivity of SKOV-3TR to doxorubicin in comparison to the MDA-MB-231 cell line. The cytotoxicity of doxorubicin in longer recovery times for MDA-MB-231 is lower than the cytotoxicity observed in SKOV-3TR (Figure 6 and Figure 7). In addition, 500 nM of siRNA concentration showed higher cytotoxicity in all the incubation and recovery time points in the 2C5-MDM-D-R nanoparticles. The cytotoxicity data show that the formulation is safe and effective.

## 5. Conclusions

We have evaluated the 2C5 antibody-modified mixed dendrimer micelles in different cell models, in addition to the models tested in our previous studies. We have successfully established the stability of the formulation in this study. Furthermore, from this study, we found that the formulations may need to be tweaked to treat different cancers. In addition, we proved that the dendrimer-based formulation is biocompatible and non-hemolytic, making it safe for animal studies. Cellular association assays performed in the study prove the 2C5 targeting capability of the formulation, and this can be further corroborated using the results on cellular internalization using Western blot analysis. The Western blot analysis showed the ability of the formulation to protect the siRNA and safely deliver the formulation inside the cells and its endosomal escape. The monolayer cytotoxicity studies prove that the 2C5-modified nanoformulation is safe and effective at 500 nM and lower concentrations of siRNA. In future, we would like to optimize the formulation further in terms of the dosage suitable for the cancer cell model being used and later test the formulation in vivo in a mice xenograft model.

## Figures and Tables

**Table 1:** Stability data of 2C5-modified MDM.

	Day 0			Day 5			Day 15			Day 20		
	Size (nm)	PdI	Zeta Potential (mV)	Size (nm)	PdI	Zeta Potential (mV)	Size (nm)	PdI	Zeta Potential (mV)	Size (nm)	PdI	Zeta Potential (mV)
2C5-modified MDM with siRNA and DOX (2C5-MDM-D-R)	165.4 ± 70.1	0.175	2.04 ± 5.96	149.8 ± 71.1	0.172	2.12 ± 7.06	160.5 ± 60.8	0.120	2.62 ± 6.04	148.4 ± 78.8	0.181	1.17 ± 5.27

## Data Availability

Not applicable.
